# ABO incompatible LRD kidney transplantation should be offered to children: results from a 33-year comparative OPTN study

**DOI:** 10.3389/ti.2026.16723

**Published:** 2026-08-17

**Authors:** Alicia Paessler, Ioannis Loukopoulos, Pankaj Chandak, Nicos Kessaris, Jelena Stojanovic

**Affiliations:** 1 Great Ormond Street Hospital for Children NHS Foundation Trust, London, United Kingdom; 2 Guy’s and St Thomas’ Hospital NHS Foundation Trust, London, United Kingdom; 3 UCL Institute of Child Health, London, United Kingdom

**Keywords:** ABO incompatible, kidney transplant, living donor, pediatric, registry

## Abstract

ABOi transplantation is a growing practice with excellent clinical outcomes. Some paediatric transplant programmes are reluctant to offer ABOi transplantation and list children on a DD waiting list. However, there are no studies directly comparing the outcomes between pediatric ABOi LD kidney transplants (LDKTx) and ABOc DD transplants (DDKTx). Data were retrieved on all pediatric kidney transplants from 1987–2020, from the United Network for Organ Sharing. Propensity score matching was used to select a control group of ABOc transplant recipients. Long term outcomes were compared between ABOi and ABOc kidney transplants and between ABOi LDKTx and ABOc DDKTx. Data were compared using chi-square test, t-test and Kaplan-Meier survival analysis. Overall, there were 70 pediatric ABOi kidney transplants meeting the study criteria. There was no significant difference in allograft (p = 0.42) and patient survival (p = 0.58) between ABOi and ABOc transplants. ABOi LDKTx had significantly lower incidence of delayed allograft function and better long-term allograft survival than ABOc DDKTx (p < 0.01, p = 0.01). ABOi transplantation has excellent long-term outcomes. ABOi LDKTx lead to better long-term outcomes than ABOc DDKTx. We recommend that ABOi transplantation from a LD should be considered prior to listing children for transplantation from a DD.

## Graphical Abstract

**Figure:**
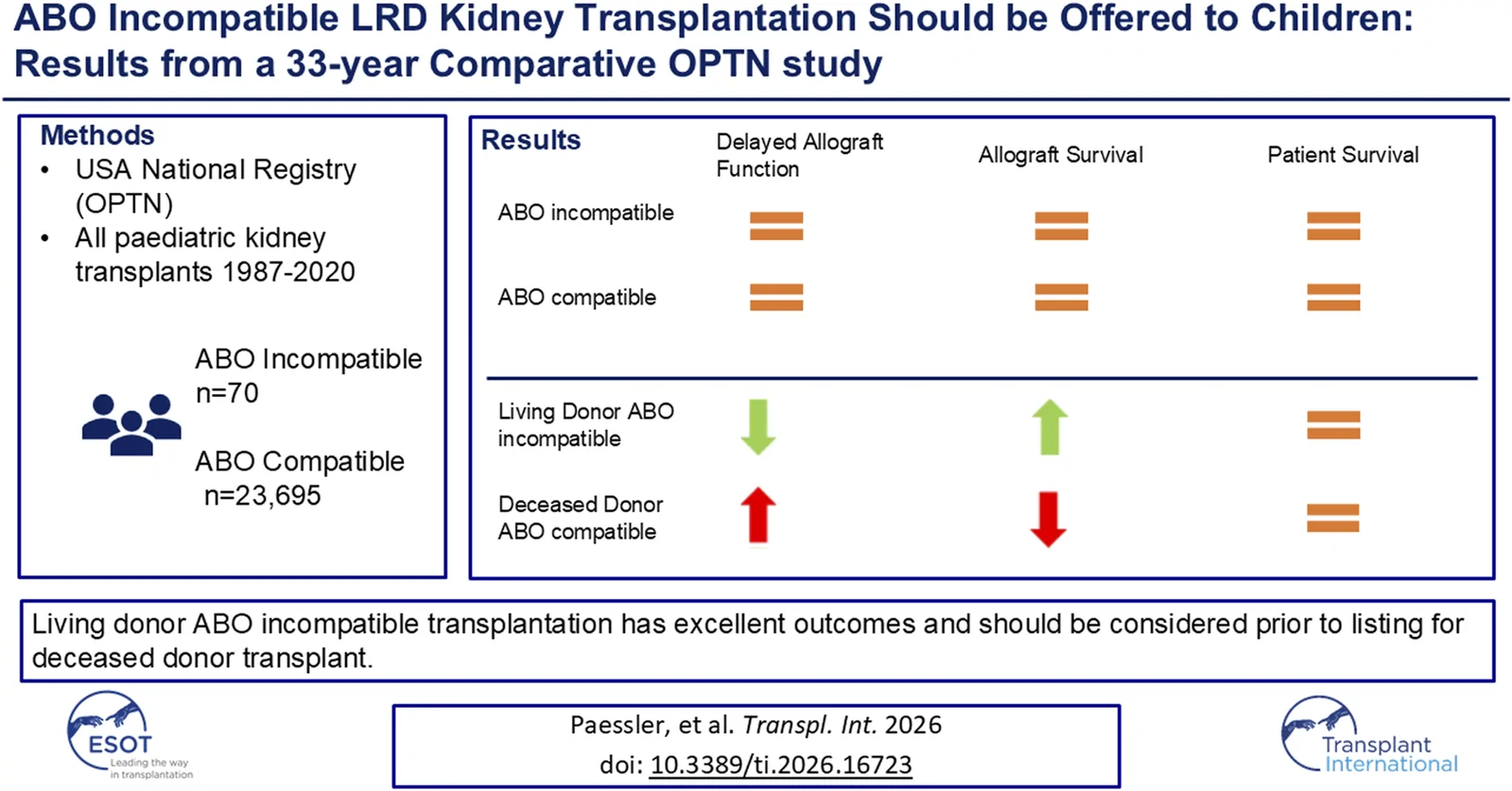

## Introduction

Shortage of organs is a major issue in pediatric renal transplantation globally. An increasing number of children with kidney failure are surviving infancy and presenting as potential kidney transplant candidates. This has created an increasing demand for donor kidneys which is reflected by the increase in pediatric patients on the waiting list (34.3% increase over 10 years) as well as an increase in the average waiting time (the number of patients waiting >1 year for a transplant increased from 55.2% to 59.7% in 10 years [1]). Despite an increasing number of children requiring a transplant, the incidence of both living and deceased donor kidney transplants have decreased by 16% and 16.1% respectively in the last 10 years [1].

The best treatment for a child with kidney failure is a pre-emptive kidney transplant from a living donor [2, 3]. Pediatric patients carry a high likelihood of requiring subsequent transplants throughout their life, so it is important that their first transplant functions for as long as possible.

Traditionally, the options for a child that has a potential living donor who is blood group incompatible, included: being listed for a deceased donor transplant or to enter a paired exchange programme. Unfortunately, paired exchange programmes are not available in all countries, and still only comprise a minority of transplants–in 2023 in the USA, only 22 children received transplants through a paired exchange programme (2.7% of overall transplant activity) [1].

A third option, which is increasingly being used, is a blood group incompatible kidney transplant. Historically, ABO incompatible (ABOi) transplants have been associated with an increased risk of rejection, infection, post-transplant lymphoproliferative disorder (PTLD) and increased graft loss [4]. Due to these risks, ABOi transplants were previously reserved for a very small subset of patients. However, there have been modern advances, largely led by Japan, in safer and more effective desensitisation techniques such as the use of B-cell depleting agents like Rituximab, and the use of immunoadsorption [5–7]. This has in turn led to improved outcomes for patients receiving ABOi transplants [5, 8] and is a potential alternative for children requiring kidney transplants who do not have a blood group compatible living donor.

While there has been an increasing amount of encouraging evidence in case reports and registry analysis from Japan [9], there is to our knowledge, no large-scale data on the outcomes of pediatric ABOi kidney transplants from another country. Therefore, our aim was to compare the short- and long-term outcomes of all pediatric ABOi kidney transplants in the USA to a propensity score matched (PSM) cohort of children receiving ABO compatible (ABOc) kidney transplants.

Furthermore, in order to try and increase the donor pool for pediatric recipients, and to equip clinicians with useful knowledge for clinical practice, we also aimed to provide evidence that may be directly applicable in clinical practice for the common clinical scenario described above in which patients have a potential living donor who is blood group incompatible. Therefore, we also carried out a sub-set analysis directly comparing the short- and long-term outcomes of ABOi kidney transplants from living donors to a PSM cohort of patients receiving ABOc kidney transplants from deceased donors.

## Materials and methods

The OPTN database is an online registry that contains all data pertaining to patient waiting lists, living and deceased organ donation, organ matching and organ transplants that have taken place in the USA since 1st October 1987 [10]. Data are added to the database at the point of listing a patient for transplant, at the point of donation and is updated at 6 months, 1-year and annually post-transplant with recipient outcome data. This database is the largest registry containing data on pediatric kidney transplants. It was chosen for this study as it would be able to provide the largest sample size for analysis. Ethical approval was not required for this study. Patient data is required as per the U.S. federal law to be included in the OPTN registry at the time of listing/donation; only de-identified data was provided so no written consent was required.

OPTN registry data for all kidney transplants in recipients under the age of 18 years in the U.S. from October 1987 until September 2020 were requested. Data retrieved from the registry included donor and recipient demographics, number of prior transplants, dialysis status at transplantation, blood group compatibility, number of HLA mismatches (at HLA-A, HLA-B and HLA-DR), primary allograft non-function, delayed allograft function, allograft survival and patient survival time. All patients’ post-transplant follow up data that were used for analysis were based on their latest data submitted to the registry in January 2021. Missing data was assumed to be missing completely at random. For each patient we only analysed data for the first kidney transplant.

All statistical analysis was carried out with IBM Statistical Package for Social Sciences (SPSS) Version 28 [11]. Propensity score matching was used to identify a control group of patients who received ABOc transplants. Propensity score matching controlled for donor type, dialysis status pre-transplant, recipient sex, recipient age, recipient ethnicity, underlying renal disease, and number of HLA mismatches. This provided a control group for comparison which was equal to the ABOi group in terms of age, sex, ethnicity, underlying disease, dialysis exposure, and HLA mismatch. Baseline demographics of all patients receiving ABOc transplants were described, as well as the baseline demographics of the PSM control group. Analysis of all post-transplant outcomes including Kaplan-Meier survival curves for allograft and patient survival was done comparing the ABOi group to the PSM ABOc group. The data in this study was not normally distributed and therefore medians and interquartile ranges were reported to describe all numerical data, frequencies and percentages were used to describe categorical data. Mann-Whitney U Test, Chi-Square Tests and Kruskal-Wallis was used for significance testing to compare groups of patients. Patient and allograft survival at 1, 3, 5 and 10 years post-transplant was estimated using Kaplan-Meier analysis and log-rank testing was used to assess comparisons. P-values, with a threshold of significance of p < 0.05 are displayed as a measure of significance. When data were used for multiple comparisons Bonferroni corrections were implemented.

A sub-set analysis was carried out comparing ABOi transplants from living donors to ABOc transplants from deceased donors. For this subset analysis a new PSM control group of patients receiving ABOc transplants from deceased donors was created which controlled for dialysis status pre-transplant, recipient sex, recipient age, recipient ethnicity, underlying renal disease, and number of HLA mismatches.

## Results

Overall, there were 23,695 pediatric ABOc kidney transplants and 70 pediatric ABOi kidney transplants. Donor and recipient blood groups of the ABOi transplants can be seen in Table 1. Although A2 to B and A2B to B transplants are widely accepted to have equivocal outcomes to traditional ABOc transplants (where anti-A titres are acceptable), these were included in this study as ABOi transplants due to the limited existing data in paediatrics. Furthermore, we do not have data on how those listed as group “A” where serotyped, i.e., if they were tested and confirmed to be A, or whether subtype was not tested and they were assumed to be A1. Nevertheless, in this cohort, A2 transplants only made up 30% of all ABOi transplants.

**TABLE 1:** Number and proportion of patients receiving ABO incompatible transplants by each donor to recipient blood group combination for all ABOi transplants and for ABOi from living donor transplants.

Donor blood type to recipient blood type	Number (all ABOI) (n = 70)	Proportion (all ABOI)	Number (ABOI-LD) (n = 50)	Proportion (ABOI-LD)
A to B	3	4.3%	2	4%
A2 to B	7	10%	3	6%
A to O	21	30%	13	26%
A2 to O	10	14.3%	9	18%
AB to A	3	4.3%	2	4%
AB to B	5	7.1%	4	8%
A2B to b	4	5.7%	2	4%
AB to O	1	1.4%	0	0%
B To O	10	14.3%	9	18%
B To A	6	8.6%	6	12%

The median recipient age did not differ significantly between the two groups – 14 (9.0–16.0) years in ABOi, and 14 (9.0–16.0) years, in the ABOc-PSM group (p = 0.48). Patient ethnicity, sex, underlying renal disease, transplant year, donor type, HLA match and dialysis status for the ABOi group, the ABOc group and PSM ABOc group can be seen in Table 2.

**TABLE 2:** Number and proportions of ABO incompatible and compatible transplants for different ethnicities, sex, underlying renal disease, eras, donor types and HLA matches. Favorable HLA match = 0, 1, 2 or 3 mismatches; unfavorable HLA match = 4, 5 or 6 mismatches. Patients with no data on ethnicity, sex, renal disease, era, dialysis status or HLA matching were excluded from analysis for their respective category.

Demographic	Sub-group	ABO incompatible (n = 70) (%)	ABO compatible (N = 23,695) (%)	P-value	ABO compatible – PSM group (n = 200) (%)	P‐value
Ethnicity	White	45 (64.3)	13,153 (55.6)	0.21	125 (62.5)	0.64
	Black	15 (21.4)	4,274 (18.0)		34 (17.0)	
	Hispanic	6 (8.6)	4,982 (21.0)		29 (14.5)	
	Asian	1 (1.4)	712 (3.0)		5 (2.5)	
	Other/Mixed	3 (4.3)	574 (2.4)		7 (3.5)	
Sex	Male	42 (60.0)	14,004 (59.1)	0.87	113 (56.5)	0.25
	Female	28 (40.0)	9,691 (40.9)		87 (43.5)	
Renal disease	Cystic	2 (6.9)	1,044 (7.6)	**<0.01**	4 (4.8)	0.99
	Obstructive/Reflux	6 (20.7)	3,221 (23.5)		21 (25.3)	
	Glomerulo-nephritis	9 (31.0)	4,424 (32.3)		27 (32.5)	
	Hypertension/Vascular	1 (3.5)	647 (4.7)		4 (4.8)	
	Hereditary/Metabolic	2 (6.9)	812 (5.9)		3 (3.6)	
	Hypoplasia/Dysplasia	6 (20.7)	2,472 (18.1)		19 (22.9)	
	Other	3 (10.3)	1,078 (7.9)	-	5 (6.0)	
Era	<1990	10 (14.3)	1,409 (6.0)	-	6 (3.0)	-
	1990–1999	24 (34.3)	6,643 (28.0)	-	79 (39.5)	-
	2000–2009	21 (30.0)	7,819 (33.0)	-	78 (39.0)	-
	2010–2019	14 (20.0)	7,372 (31.1)	-	36 (18.0)	-
	2020 >	1 (1.4)	452 (1.9)	-	1 (0.5)	-
Donor type	Living	50 (71.4)	10,588 (44.7)	**<0.01**	136 (68.0)	0.28
	DBD	19 (27.1)	12,754 (53.8)		64 (32.0)	
	DCD	1 (1.5)	353 (1.5)		0 (0.0)	
HLA match	Favorable	47 (69.1)	11,646 (49.9)	**<0.01**	142 (71.0)	0.76
	Unfavorable	21 (30.9)	11,714 (50.1)		58 (29.0)	
Dialysis status	Pre-emptive	21 (30.0)	5,500 (23.2)	0.17	60 (30.0)	1.0
	On dialysis	49 (70.0)	18,195 (76.8)		40 (70.0)	

Bold values indicate statistical significance.

### Post-transplant outcomes

The median follow up time post-transplant for the ABOi group was 6.3 years (3.0–12.1 years). There was no significant difference in the incidence of delayed allograft function (DGF) between ABOi and ABOc transplants: n = 4, 5.7% in ABOi and n = 16, 8.0% in ABOc (p = 0.89). The same findings were seen in the incidence of primary non-function: n = 1, 1.4% in ABOi and n = 1, 0.5% in ABOc (p = 0.77). There were no cases of renal vein thrombosis in ABOi transplants whilst in the ABOc group five children had thrombosis (2.5%, p = 0.60).

**FIGURE 1:**
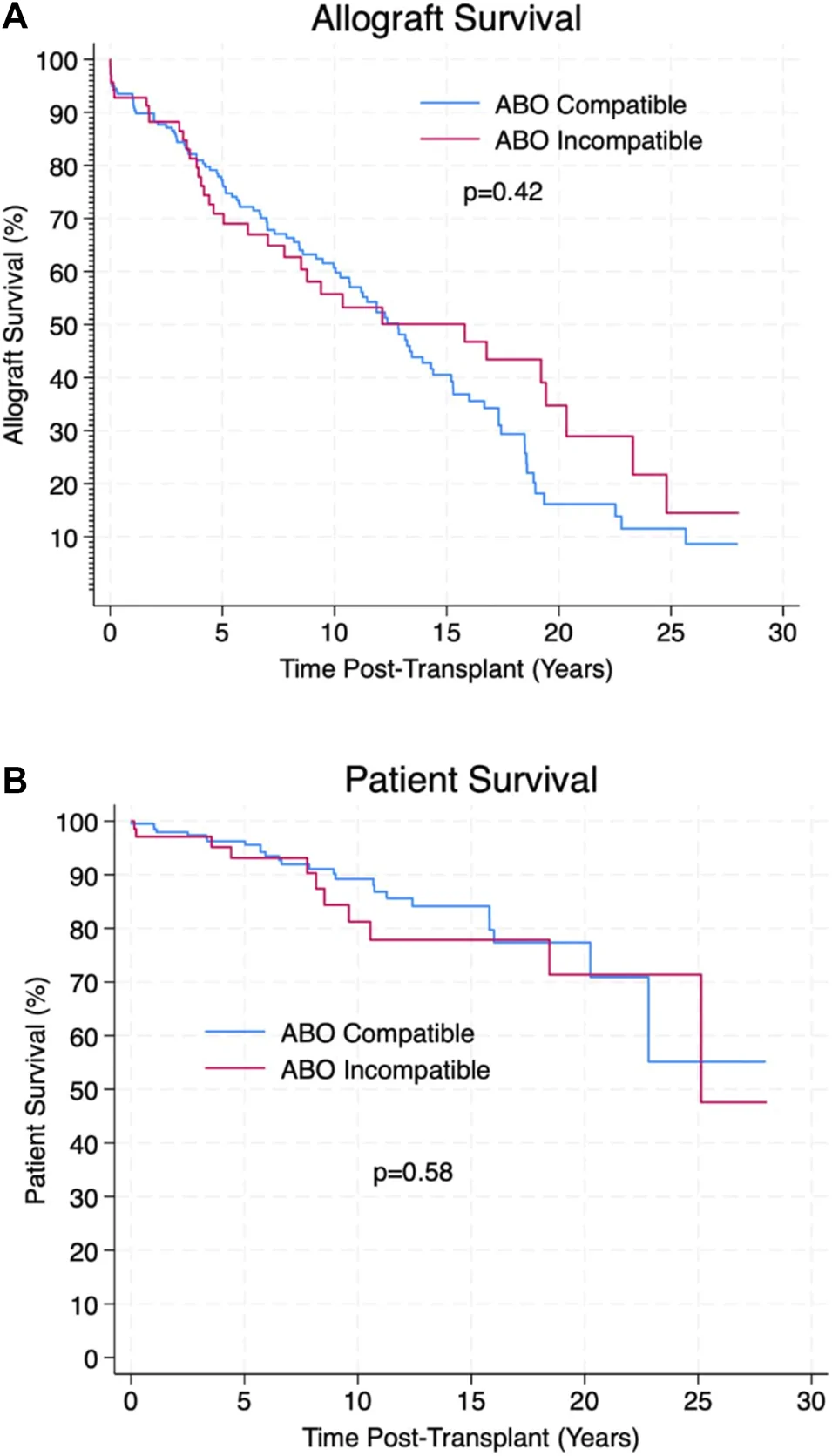
**(A,B)** Estimated Kaplan-Meier graft and patient survival (respectively) post-transplant for ABOi and ABOc transplants.

**FIGURE 2:**
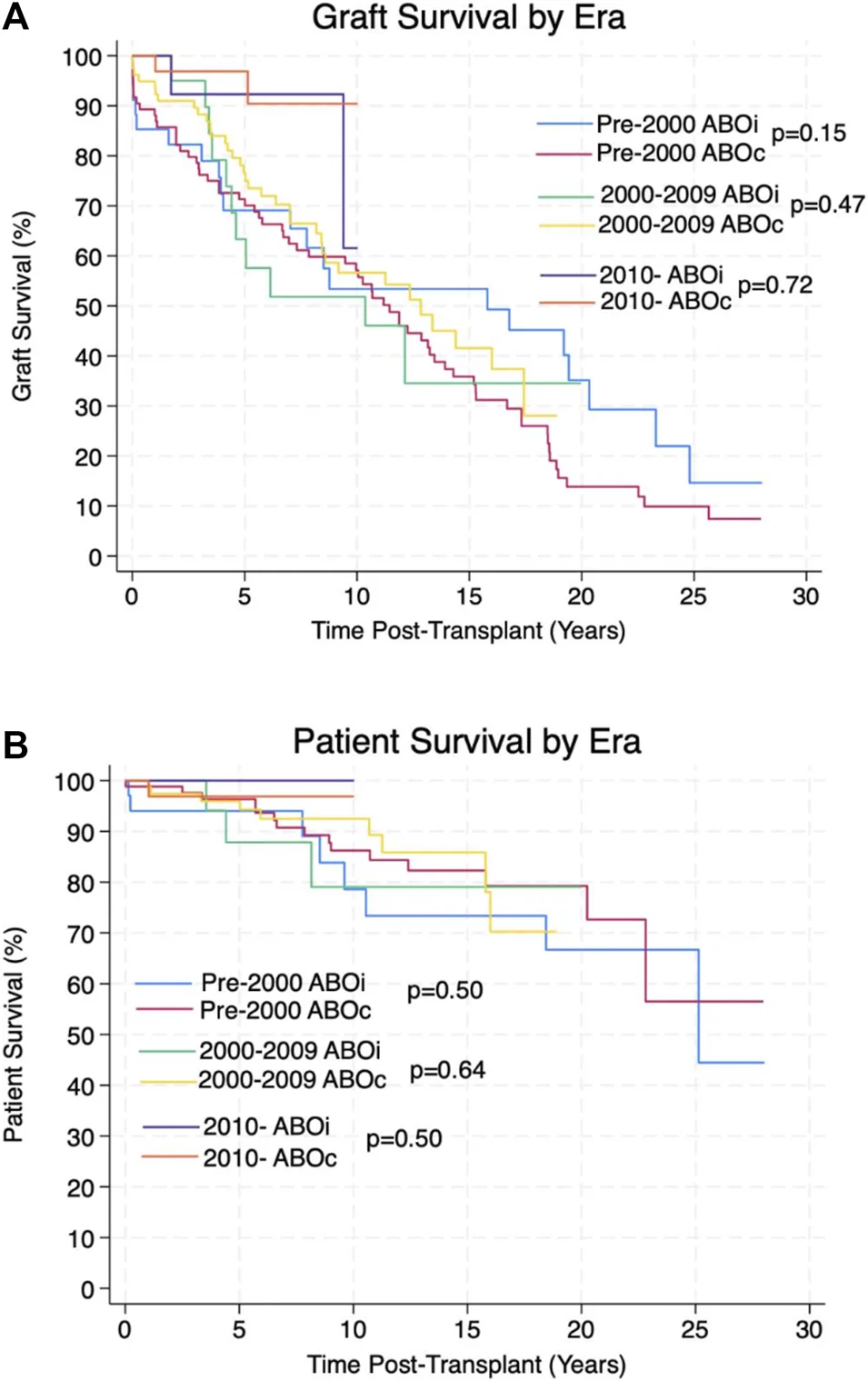
**(A,B)** Estimated Kaplan-Meier graft and patient survival (respectively) post-transplant for patients with ABOi and ABOc across different eras.

There was no significant difference in both allograft and patient survival between ABOi and ABOc transplants. 1, 3, 5 and 10 years allograft and patient survival is summarized in Table 3 and survival curves can be seen in Figures 1A,B. Patient and allograft survival across different eras of transplantation are summarized in Table 4 and Figures 2A,B.

**TABLE 3:** Overall estimated Kaplan-Meier graft and patient survival for ABOi and ABOc (PSM group) transplants at 1, 3, 5 and 10 years post-transplant. The ABOc group refers to the propensity-score-matched group.

Survival	Patient group	1 year	3 years	5 years	10 years	P-value
Allograft survival	ABOi (n = 70)	93%	88%	69%	56%	0.42
	Number at risk	63	54	38	23	
	ABOc (n = 200)	93%	84%	77%	60%	
	Number at risk	179	152	123	69	
Patient survival	ABOi (n = 70)	97%	97%	93%	81%	0.58
	Number at risk	65	54	42	25	
	ABOc (n = 200)	99%	97%	96%	89%	
	Number at risk	193	172	147	83	

**TABLE 4:** Estimated Kaplan-Meier graft and patient survival for patients receiving ABOi transplants ABOi transplants across different timepoints at 1, 3, 5 and 10 years post-transplant. The ABOc group refers to the propensity-score-matched group.

Survival	Era		1 year	3 years	5 years	10 years	P-value
Graft Survival	Pre-2000	ABOi (n = 34)	85%	82%	69%	53%	0.15
		Number at Risk	29	26	20	13	
		ABOc (n = 85)	87%	75%	70%	57%	
		Number at Risk	74	64	57	42	
	2000–2009	ABOi (n = 21)	95%	90%	58%	46%	0.47
		Number at Risk	20	18	11	9	
		ABOc (n = 78)	94%	88%	77%	57%	
		Number at Risk	73	64	51	26	
	2010 - current	ABOi (n = 15)	100%	92%	92%	61%	0.10
		Number at Risk	14	10	7	1	
		ABOc (n = 37)	100%	97%	90%	90%	
		Number at Risk	33	24	15	1	
Patient Survival	Pre-2000	ABOi (n = 34)	94%	94%	94%	73%	0.50
		Number at Risk	31	26	23	15	
		ABOc (n = 85)	99%	98%	96%	86%	
		Number at Risk	84	79	73	51	
	2000–2009	ABOi (n = 21)	100%	100%	88%	79%	0.54
		Number at Risk	20	18	12	9	
		ABOc (n = 78)	99%	97%	96%	92%	
		Number at Risk	77	69	59	31	
	2010 - current	ABOi (n = 15)	100%	100%	100%	100%	0.50
		Number at Risk	14	10	7	1	
		ABOc (n = 37)	100%	97%	97%	97%	
		Number at Risk	33	24	15	1	

Further sub-set analysis was carried out to compare the outcomes between A, A2, B and AB incompatible transplants and found no significant difference in allograft survival (p = 0.43) nor patient survival (p = 0.71).

### Impact of donor type

Outcomes were also compared between patients who received ABOi transplants from living donors (n = 50) and a PSM group of patients who received ABOc transplants from deceased donors (n = 144).

Patient receiving ABOi transplants from living donors experienced significantly less DGF than patients receiving ABOc transplants from deceased donors–n = 1, 2% vs. n = 23, 16.0% (p < 0.01). However, there was no significant difference in incidence of primary non-function–n = 1, 2% in ABOi transplants from living donors and n = 4, 2.8% in ABOc transplants from deceased donors (p = 0.76).

**FIGURE 3:**
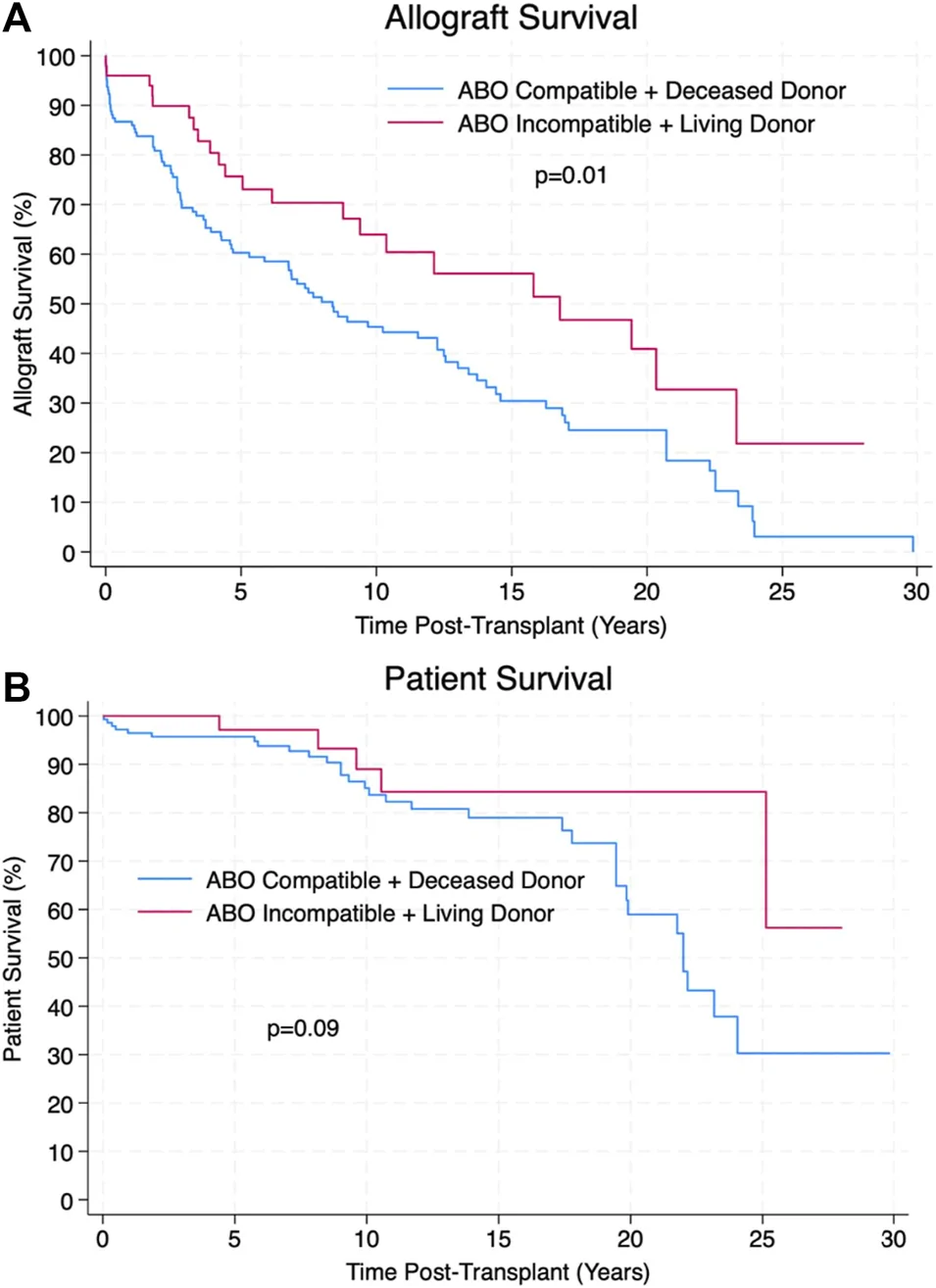
**(A,B)** Estimated Kaplan-Meier graft and patient survival (respectively) post-transplant for patients with ABOi transplants from living donors and for patients with ABOc transplants from deceased donors.

Analysis showed that patients receiving ABOi transplants from living donors had significantly better allograft survival than patients receiving ABOc transplants from deceased donors, although there was no difference in patient survival between the two groups. 1, 3, 5, and 10 years allograft and patient survival for these patients is summarized in Table 5 and survival curves can be seen in Figures 3A,B. The same trend continued after 10 years as seen in Figures 3A,B.

**TABLE 5:** Estimated Kaplan-Meier graft and patient survival for patients receiving ABOi transplants from living donors, ABOc transplants from deceased donors (PSM Group) and ABOc transplants from living donors (PSM Group) at 1, 3, 5 and 10 years post-transplant.

Survival	Patient group	1 year	3 years	5 years	10 years	P-value
Graft Survival	ABOi – Living Donor (n = 50)	96%	90%	73%	64%	ABOi-LD vs. ABOc-DD p = 0.01 ABOi-LD vs. ABOc-LD p = 0.42
	Number at Risk	47	40	29	19	
	ABOc – Deceased donor (n = 64)	86%	69%	59%	44%	
	Number at Risk	54	46	39	20	
	ABOc – Living donor (n = 136)	94%	86%	76%	65%	
	Number at Risk	125	106	84	49	
Patient survival	ABOi – Living donor (n = 50)	100%	100%	97%	89%	ABOi-LD vs. ABOc-DD p = 0.09 ABOi-LD vs. ABOc-LD p = 0.55
	Number at Risk	48	40	31	20	
	ABOc – Deceased donor (n = 64)	97%	96%	96%	84%	
	Number at Risk	62	52	45	27	
	ABOc – Living donor (n = 136)	99%	98%	95%	90%	
	Number at Risk	132	120	102	56	

A further sub-analysis was carried out to analyse just living donor transplants to assess whether controlling for donor type would reveal a difference in outcomes from ABOi transplants. This found there was no significant difference in the incidence of DGF (p = 0.13), incidence of primary non-function (p = 0.46), allograft survival (p = 0.42) nor patient survival (p = 0.55).

## Discussion

We present the first study analysing the outcomes of all ABOi pediatric kidney transplants to date in one of the countries with the largest transplant programme–the USA and the first to analyse a direct comparison between ABOi kidney transplants from living donors and ABOc kidney transplants from deceased donors. We show that ABOi pediatric kidney transplants have equitable short and long-term outcomes to ABOc pediatric kidney transplants. We also show that on direct comparison, ABOi transplants from living donors have lower incidence of delayed allograft function and better allograft survival than ABOc transplants from deceased donors.

Our findings are consistent with those reported in the literature. A Japanese study of Kidney Transplant Registry showed that there was no difference in graft survival or patient survival between ABOi and ABOc in 102 pediatric ABOi kidney transplants [5]. Encouragingly, this study also found no difference in infectious complications between ABOi and ABOc recipients [5]. Additionally, a UK study with 11 patients showed no difference between ABOi and ABOc with respect to graft function and acute rejection [7]. Another study with 52 patients had similar allograft and patient survival rates to our study and also did not show any difference compared to ABOc transplants [12]. They also showed that ABOi transplants did have higher incidence of acute rejection, however, after the addition of Mycophenolate to the standard immunosuppression regime for these patients, this improved significantly [12].

Recently there have been changes in the way that ABOi transplants have been facilitated with a lot of variation in desensitisation techniques. Previously, patients underwent splenectomies prior to transplant in addition to plasmapheresis which posed higher risks. Currently the accepted strategy used by many centers involves plasmapheresis or immunoadsorption pre-operatively to reduce antibody titres. Many centers aim to reduce titres to <1/8 [5, 7, 12, 13], however one study has also shown that reducing antibody titres to <1/32 led to good outcomes [9]. Current evidence has found that using this strategy has not led to any increase in infectious complications [6].

Unfortunately our study did not have data on the immunosuppression used so we cannot comment on whether these patients had escalated immunosuppression, however, there are some case reports of safe ABOi transplantation with good outcomes without any additional desensitization in patients who have antibody titres of <1/8 at baseline [6, 7]. Another case report from South Africa discussed a patient with titres <1/8 who was safely transplanted with standard immunosuppression and only one dose of Rituximab prior to transplant which shows that ABOi transplantation may be possible even in some middle-/low-income settings [14]. This suggests that patients with antibody titres <1/8 may potentially also be considered for ABOi transplantation from deceased donors, as no additional pre-transplant desensitisation may be needed. One study simulated a kidney allocation scheme that would allow children on the deceased donor waiting list with antibody titres of <1/16 to be allocated ABOi transplants and found that this strategy could potentially reduce waiting times and lead to a 2.2% increase in transplants while also increasing the number of children receiving transplants with 0 HLA mismatches at A, B and DR [15]. However, it is also important to note that reporting titre measuring methods is crucial as there is significant variability in the methods leading to a lack of standardization and significant variability in results [16]. We would encourage that further published studies describe the methods used for titre measurement and where possible that these be standardised (with for example, new assays based on single-antigen bead technology [17]) for the purpose of making transplant decisions.

Another advantage of ABOi transplants from living donors is the benefit of planned surgery which can occur in a more controlled environment. For example, when transplanting children with inherited metabolic disorders, ensuring clinical stability prior to transplant is crucial [18], so planning for a living donor ABOi transplant over a deceased donor ABOc transplant allows the clinical team more control and can ensure the patient is transplanted in optimal circumstances.

An alternative to ABOi transplantation is the use of paired donation schemes. There is significant evidence that paired donation and non-paired living donation provides the same outcomes, although this approach continues to be underutilized in the USA in comparison to other countries [19–21]. Paired donation is an additional opportunity for patients to benefit from living donation. Given the perceived risk and the thus far limited evidence and somewhat limited use of ABOi transplantation in children, paired exchange schemes provide an excellent opportunity. Unfortunately, there is a only a limited number of centres in the USA that participate in the scheme so this does limit the potential opportunities it may provide and effort should be focused on expanding the scheme to more centers [19]. Where patients have blood group incompatible living donors and lack of access to a paired scheme, ABOi transplantation should be considered prior to deceased donation.

Our study has several advantages including a large sample size of 70 ABOi paediatric transplants over a 30 year period with a median follow up of 6 years. While 70 ABOi transplants may not be a large sample size compared to some adult studies and may limit some of the statistical conclusions, it is one of the largest sample sizes to date within paediatrics. Encouragingly, when outcomes were stratified by era of transplantation to try and control for changes in practice in transplantation over time, our results continued to show the same findings–that ABOi transplants had equitable outcomes to ABOc transplants. Furthermore, using propensity score matching allows this study to control for many potential confounders such as year of transplantation, age, dialysis status, underlying disease, HLA matching etc. and allows the results attributed to the ABO compatibility to be more reliable.

Our study is limited by having no data on pre-transplant antibody titres, titre-measurement methods, centre variability in titre level acceptability (particularly for A2-incompatible transplants) nor cPRA or which desensitization techniques used. This adds significant variability to the degree of mismatch between the transplants and also variability between desensitisation protocols which can make long-term outcomes more difficult to generalize. ABOi transplants typically are done with different induction therapies compared to ABOc; our registry data does not hold this data. Therefore we were not able to control for this in the propensity score matching, nor present data on how this theoretical difference in immunosuppression may have influenced transplant and patient outcomes. Our lack of data on these is an important factor to consider when interpreting our results. We also do not have data on the incidence of rejection or infectious complications. Nevertheless, as our results do not show a difference in long-term allograft and patient survival between ABOi and ABOc transplants; any potential differences in rejection or infectious complications are not significant enough to impact long-term outcomes which is very reassuring. While using registry data allows us to have a large enough sample size to carry out analysis on outcomes, it does also mean that our data is limited by what variables are held by the registry and relies on the accuracy of the data that is submitted to the registry. For example, we only have data on HLA matching at HLA-A, HLA-B and HLA-DR; it is possible that mismatches at other loci which may have impacts on outcomes could be confounding the results. Additionally we also did not have data on cPRA of the two cohorts either.

Another important consideration is that OPTN largely relies on the lectin method to determine ABO, whereas there are now new molecular methods to determine ABO more accurately [22]. The lectin method is particularly less accurate in infants and neonates [23] so there’s a possibility that some of the reported ABO types for the younger recipients and donors is not accurate and so some of the transplants reports as ABOi are not incompatible and equally some reported as compatible may in fact be ABOi. Unfortunately, we do not have data on how frequently ABO mistyping occurs due to using serotyping as opposed to phenotyping in the OPTN. Some studies have found that discrepancies in ABO compatibility between sero- and genotyping can occur in up to 42% of patients so it is crucial that further research is carried out using the more up to date molecular methods of ABO classification.

Due to the difficulty in accurately identifying A subgroups, amongst other factors, there has been limited uptake in A2-incompatible transplants. The positive outcomes of A2-incompatible transplants have been described in adults, and there appears to be an increased acceptability of this type of incompatible transplants [24]. However, studies have shown that when using more advanced genotyping methods, up to 55% of patients believed to be A1 may actually be A2, and using more up to date molecular ABO classifications methods may facilitate more transplants [17]. It is crucial that these newer methods should be brought into every day clinical practice and inform transplant decisions.

Furthermore, we also do not have data on which centers carried out these transplants and so it is possible that there are some “center effects” contributing to the difference in outcomes that we were not able to control for in the propensity matching. However, we do recommend that as ABOi transplantation becomes more common practice, it should initially be carried out at or in collaboration with centers who have experience in immunologically complex transplantation.

## Conclusion

In conclusion, our registry analysis and propensity score matched study spanning over 30 years, has found that pediatric ABOi kidney transplants can have excellent outcomes that do not differ significantly from ABOc kidney transplants. More importantly, we also show that ABOi transplants from living donors showed better long-term outcomes than ABOc transplants from deceased donors. While we cannot comment on induction therapies and immunosuppression, rejection or infections, and the lack of this data should be kept in mind when interpreting our results, long-term graft and patient survival does not differ significantly which suggests that these transplants are a viable option that should be considered for children requiring transplantation.

In patients who have a potential living donor who is blood group incompatible and either do not have access to or have not been successfully paired in an exchange programme, one may consider proceeding with ABOi transplantation prior to listing for deceased donor transplantation. However, we recommend that these transplants should initially be done in collaboration with high-volume centres who have experience in immunologically complex transplants. Prospective studies comparing ABOi transplantation with paired exchange transplants and deceased donor transplants are necessary to further support the evidence given in our study and determine the best approach for these patients.

## Data Availability

Publicly available datasets were analyzed in this study. This data can be found here: https://www.hrsa.gov/optn/data/data-reports.
